# Branch-like enhancement on contrast enhanced MRI is a specific finding of cerebellar lymphoma compared with other pathologies

**DOI:** 10.1038/s41598-022-07581-x

**Published:** 2022-03-04

**Authors:** Kota Yokoyama, Jun Oyama, Junichi Tsuchiya, Jun Karakama, Kaoru Tamura, Motoki Inaji, Yoji Tanaka, Daisuke Kobayashi, Taketoshi Maehara, Ukihide Tateishi

**Affiliations:** 1 Department of Diagnostic Radiology, Tokyo Medical and Dental University, 1-5-45, Yushima, Bunkyo-ku, Tokyo, 113-8519 Japan; 2 Department of Neurosurgery, Tokyo Medical and Dental University, Tokyo, Japan; 3 Department of Pathology, Tokyo Medical and Dental University, Tokyo, Japan

**Keywords:** Neurology, Oncology, Lymphoma, Cancer imaging, Diagnostic markers, CNS cancer

## Abstract

Branch-like enhancement (BLE) on contrast-enhanced (CE) magnetic resonance imaging (MRI) was found to be effective in differentiating primary central nervous system lymphoma (PCNSL) from high-grade glioma (HGG) in the cerebellum. However, whether it can be applied to assessments of secondary central nervous system lymphoma (SCNSL), or other cerebellar lesions is unknown. Hence, we retrospectively reviewed cerebellar masses to investigate the use of BLE in differentiating cerebellar lymphoma (CL), both primary and secondary, from other lesions. Two reviewers qualitatively evaluated the presence and degree of BLE on CE-T1 weighted imaging (T1WI). If multiple views were available, we determined the view in which BLE was the most visible. Seventy-five patients with the following pathologies were identified:17 patients with CL, 30 patients with metastasis, 12 patients with hemangioblastoma, 9 patients with HGG, and 7 patients with others. Twelve patients presented with PCNSL and five with SCNSL. Of 17 patients with CL, 15 (88%) had BLE, whereas three (5%) out of 58 patients in the non-CL group showed BLE. In patients who underwent three-dimensional-CE-T1WI, BLE was the most visible on the sagittal image. In conclusion, BLE is a highly specific finding for CL and the sagittal image is important in evaluating this finding.

## Introduction

Lymphoma accounts for about 5–6% of all malignancies^1^. Malignant lymphoma with central nervous system (CNS) involvement is a rare condition that has a poor prognosis. The incidence of CNS involvement in non-Hodgkin lymphoma ranges from 2.2 to 27% in different studies^2^. It may be secondary or may arise from the brain parenchyma itself, and this is referred to as primary central nervous system lymphoma (PCNSL). PCNSL accounts for 4% of newly diagnosed brain tumors and 4–6% of all extra-nodal lymphomas, with an incidence of 0.4–0.5/100,000 per year^3,4^. Secondary CNS lymphoma (SCNSL) is defined as secondary CNS involvement in patients with systemic lymphoma^5^. It can be classified as systemic lymphomas with CNS involvement at the time of diagnosis or relapse or progression and isolated CNS relapse despite systemic remission^6^. SCNSL can occur in approximately 5% of patients with diffuse large B cell lymphoma (DLBCL), which has a poor prognosis, with an overall survival of only 3.9–7.2 months^7,8^. DLBCL is the most common subtype, which accounts for more than 90% of all CNS lymphoma cases^9^. PCNSL or SCNSL is commonly diagnosed based on the combination of clinical presentation, radiological manifestations, and cerebrospinal fluid test findings^5^. Meanwhile, contrast-enhanced (CE) MRI plays an important role in diagnosis and response assessments^10–12^. In MRI, CNS lymphoma commonly presents as a supratentorial single infiltrative intracranial mass. However, it may also manifest as multiple masses. The frontal lobe (20–49%), basal ganglia and/or thalamus (15–32%), and corpus callosum (15–19%) are commonly involved^13–15^. Infratentorial parenchymal involvement is less frequent (9–13%) and almost exclusively observed in SCNSL^16^. The difference between PCNSL and SCNSL is rarely investigated. Some studies have shown that SCNSL typically presents with leptomeningeal involvement^16^, and deep gray matter and infratentorial involvement are less common in SCNSL than in PCNSL^17^. Some studies have shown no significant difference between PCNSL and SCNSL, both predominantly presenting as multiple lesions with consistent supratentorial white matter involvement^18,19^. The first CE-MRI case report of CL showed a linear streak enhancement extending from the cerebellar mass peripherally into the white matter of the cerebellar hemisphere. This finding was considered unusual in patients with CL at the time of the study^20^. However, a recent research study compared cerebellar PCNSL with high-grade glioma (HGG) showed that the linear streak enhancement, referred to as branch-like enhancement (BLE), was effective in differentiating PCNSL from HGG^21^. The diagnosis of CNSL can be challenging because of its extensively variable appearance on imaging and whether the finding can be applied to assessments of secondary central nervous system lymphoma (SCNSL), or other cerebellar lesions is unknown. Therefore, this study aimed to investigate the frequency and application of BLE in differentiating CL from other mass lesions.

## Materials and methods

### Participants and data extraction

From January 1, 2000, to March 31, 2021, we retrospectively reviewed our radiology information system and searched all reports using the following keywords: “cerebellar OR cerebellum OR infratentorial OR posterior fossa” AND “lymphoma OR mass OR tumor OR lesion OR enhancement.” Reports and related images were screened to identify patients with possible CL (cerebellar mass or abnormal-enhancing lesion) who underwent CE-MRI. We excluded patients without CE-MRI data at the time of diagnosis (before biopsy or operation), those with cancer who presented with only small nodular lesions, those with extra-axial lesions without parenchymal invasion, and those without any cerebellar lesions. We reviewed the medical records and the final diagnoses of these patients. Then, patients without a definite final diagnosis were excluded. For patients with a solitary cerebellar mass, a pathological diagnosis was mandatory. However, a clinical diagnosis of secondary cerebellar lesions including SCNSL, or metastasis was acceptable. Then, clinical data, such as age, sex, and clinical course, were recorded.

### MRI protocol

The routine MRI protocol at our hospital is previously described^22^. In the present study, CE-T1WI was obtained after bolus administration of the contrast agent Gd-DTPA (Magnevist, Bayer-Schering, Berlin, Germany) at 0.1 mmol/kg body weight (0.2 ml/kg). Two-dimensional (2D) CE-T1WI was acquired with axial and coronal or sagittal T1-weighted inversion recovery 2D TSE images (TR/TE/TI/NEX: 2000–3800 ms/7.8–13.3 ms/860–1300 ms/1–2; n ¼ 657) with a section thickness of 5 mm, an intersection gap of 1–1.5 mm, FOV 210 mm, and matrix 512–256 × 360–192 were used for the acquisition. Three-dimensional (3D) CE-T1WI was obtained with CUBE (TR/TE/NEX: 600 ms/9.9–11.8 ms/1) or BRAVO (TR/TE/TI/NEX: 6.0–7.0 ms/2.5–3.0 ms/400–450 ms/1) or 3DMOVX (TR/TE/NEX: 14 ms/5.5 ms/1) or isoFSE (TR/TE/TI/NEX: 450 ms/4.0–6.2 ms/3.0 ms/1) or SPGR (TR/TE/ TI/NEX: 7–9.7 ms/2.8–4 ms/450 ms/1) with a section thickness of 1–2 mm, an intersection gap of 0–1 mm, a FOV of 210 mm, and a matrix of 288–256 × 288–256.

### Image analyses

The main location of the cerebellar lesions and whether the patient had a single lesion or multiple lesions were recorded. Only cerebellar lesions were evaluated in the analysis. Two reviewers (a neuroradiologist with 12 years of experience and a general radiologist with 13 years of experience) independently reviewed the CE-T1WI findings of patients with cerebellar lesions, and they were not informed about the final diagnoses. The presence and degree of BLE were qualitatively evaluated in four stages, as follows: none, mild, moderate, and clear. We recorded whether 3D or 2D CE-T1WI was performed. If multiple views (axial, sagittal, and coronal) were available, BLE was evaluated on each view. The view in which the findings were the most visible was determined based on a consensus decision. Although not required, a subject extractor (third reviewer) was considered to settle any disagreement.

In the secondary analysis, we qualitatively evaluated other available imaging findings for the presence of the following: clear hyperintensity on DWI and a low ADC, which was described as restricted diffusion, streak-like edema^21^, a cystic component, and a signal indicating hemorrhage on T1WI or T2WI. In addition, if images were obtained simultaneously, findings including a high-density area on a CT scan and high activity on FDG PET or PET/CT were recorded.

As a comparison within the group, we examined whether there were differences in the clinical and imaging features of PCNSL and SCNSL.

### Statistical analyses

MedCalc (version 11.6; MedCalc Software) and the Statistical Package for the Social Sciences (version 27; IBM Corp.) were used for statistical analyses. One-way analysis of variance (ANOVA) and post hoc pairwise comparisons with the Tukey–Kramer test were used to examine the differences between the CL and the other groups, with p < 0.05 considered statistically significant. The inter-reader agreement for BLE assessment in each view was evaluated using weighted kappa.

### Ethical approval

We declare that all procedures performed in studies involving human participants were in accordance with the ethical standards of the institutional and/or national research committee and with the 1964 Helsinki declaration and its later amendments or comparable ethical standards.

### Informed consent

For the retrospective study, which ensured anonymity, informed consent were not waived, but were posted on the website, and those who could not consent were asked to opt out, which was approved by the Institutional Review Board of Tokyo Medical and Dental University ((M2021-095 (19 July, 2021)).

## Results

### Characteristics of patients

**Figure 1:**
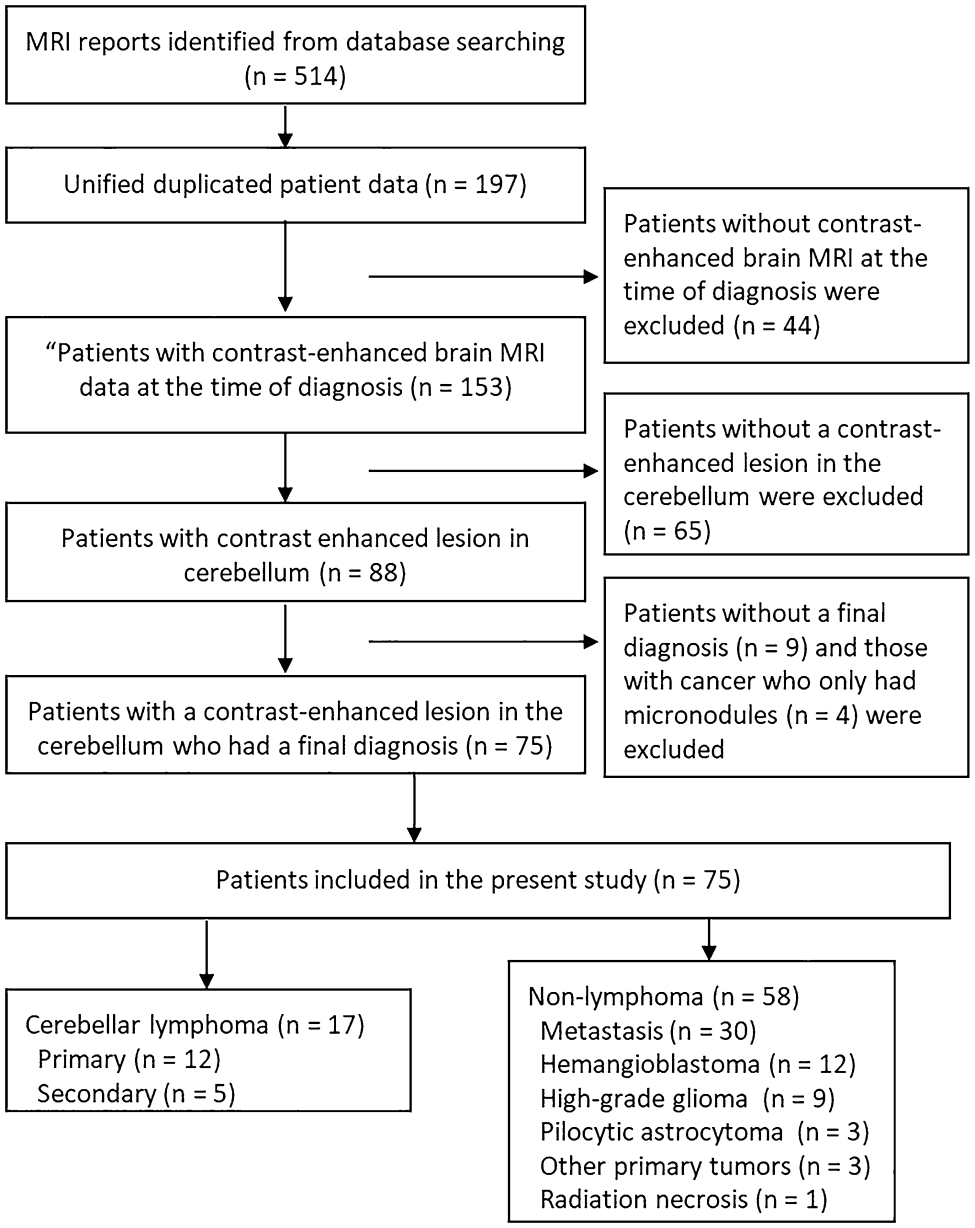
Flowchart of patient inclusion and exclusion.

In total, 75 patients for possible CL presented with cerebellar-enhancing lesions. Figure 1 shows the flowchart of patient exclusion and inclusion. Table 1 depicts the characteristics of patients. In total, 17 patients presented with CL, 30 with metastasis, 12 with hemangioblastoma, 9 with HGG, 3 with pilocytic astrocytoma, 2 with medulloblastoma, 1 with ependymoma, and 1 with radiation necrosis. The median age in CL was 64.1 (range: 9–79) years, and four (23.5%) patients were women. Twelve and five patients had PCNSL and SCNSL, respectively. In total, 11 of 12 patients with PCNSL had DLBCL, which was diagnosed by evaluating biopsy or operative specimens. One patient had primary vitreoretinal lymphoma with cerebellar invasion at the time of diagnosis. Four patients with SCNSL had a history of treatment for systemic DLBCL without CNS involvement. One patient with T-cell acute lymphoblastic leukemia presenting as a cerebellar mass was in the post-transplant state. The lesion was diagnosed as post-transplant lymphoproliferative disorder after a biopsy revealed that it was similar to EBV-positive DLBCL.

**Table 1:** Characteristics of patients.

	Cerebellar lymphoma	Metastasis	Hemangioblastoma	High-grade glioma	Others
N	17	30	12	9	7
Age	64.1 ± 15.3 (9–79)	63.3 ± 11.0 (36–88)	50.0 ± 18.0 (25–71)	46.6 ± 17.3 (24–77)	25.4 ± 24.7 (3–70)
Female:male	04:13	11:19	7:5	3:6	1:6
Background	PCNSL (12), SCNSL (5)	Colon (7), NSCLC (6), SCLC (5), breast (4), oral (2), esphogeal (2), gastric (2), ovarian (1), bladder (1)		GBM (3), AA (5), DMG (1)	Pilocytic astrocytoma (3), medulloblastoma (2), ependymoma (1), radiation necrosis (1)

*N* number, *PCNSL* primary central nervous system lymphoma, *SCNSL* secondary central nervous system lymphoma, *NSCLC* non-small cell lung cancer, *SCLC* small cell lung cancer, *GBM* glioblastoma, *AA* anaplastic astrocytoma, *DMG* diffuse midline glioma.

### BLE on CE-MRI

**Figure 2:**
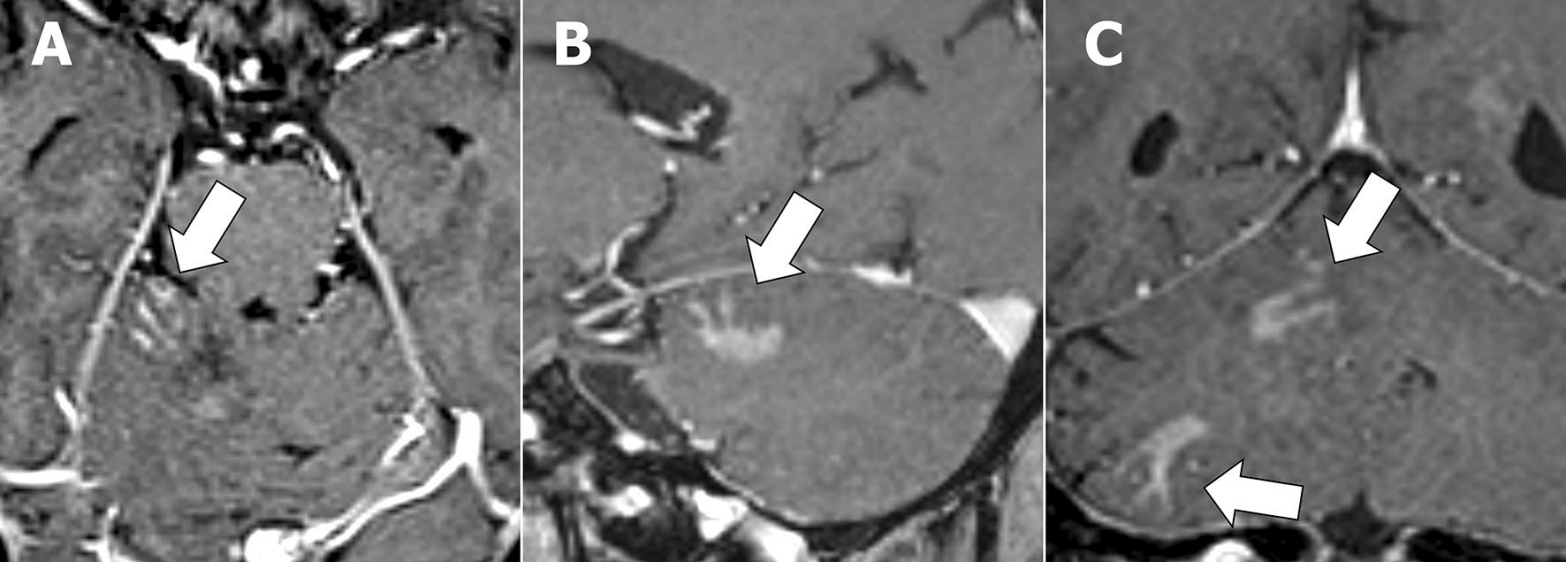
A 68-year-old male patient with secondary central nervous system lymphoma. He had a history of treatment for systemic lymphoma and had been in complete remission for 7 years. Clear branch-like enhancements (white arrows) were observed on axial (**A**), sagittal (**B**), and coronal (**C**) three-dimensional contrast-enhanced T1WI.

**Figure 3:**
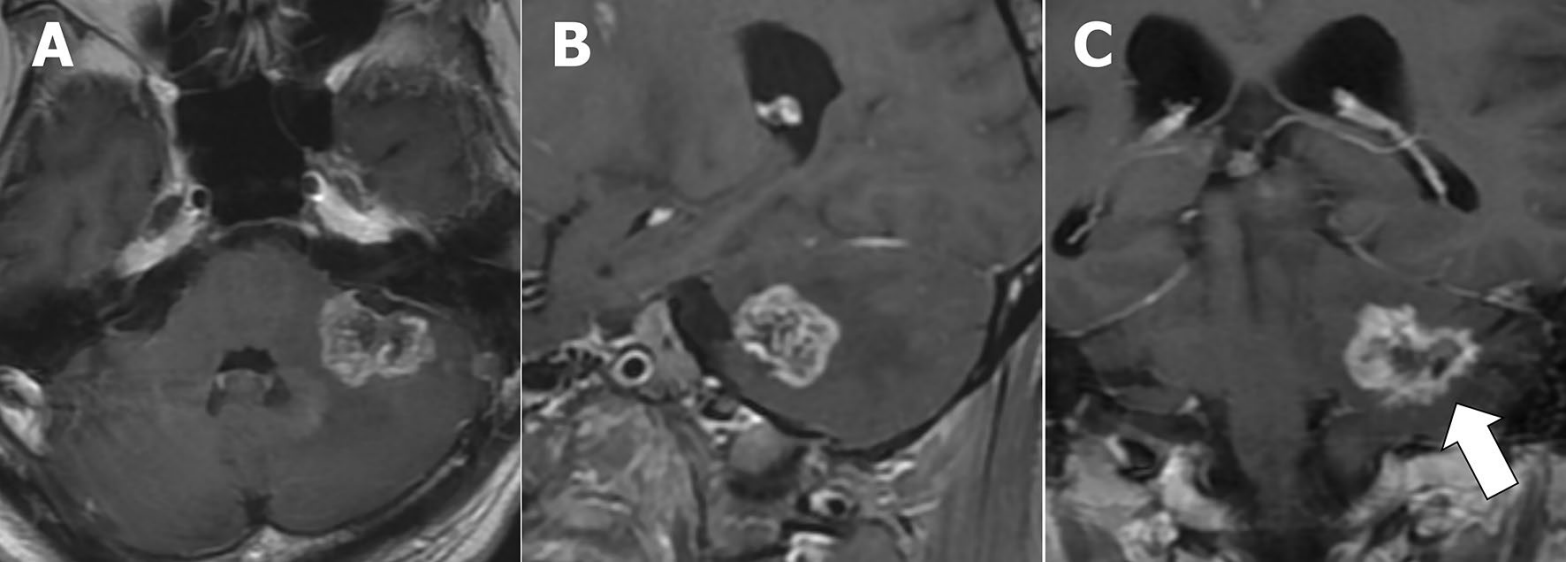
A 45-year-old male patient with diffuse midline glioma. No branch-like enhancement was observed in the axial (**A**) and sagittal (**B**) images. Meanwhile, the coronal image (**C**) showed a mild branch-like enhancement (white arrow).

**Figure 4:**
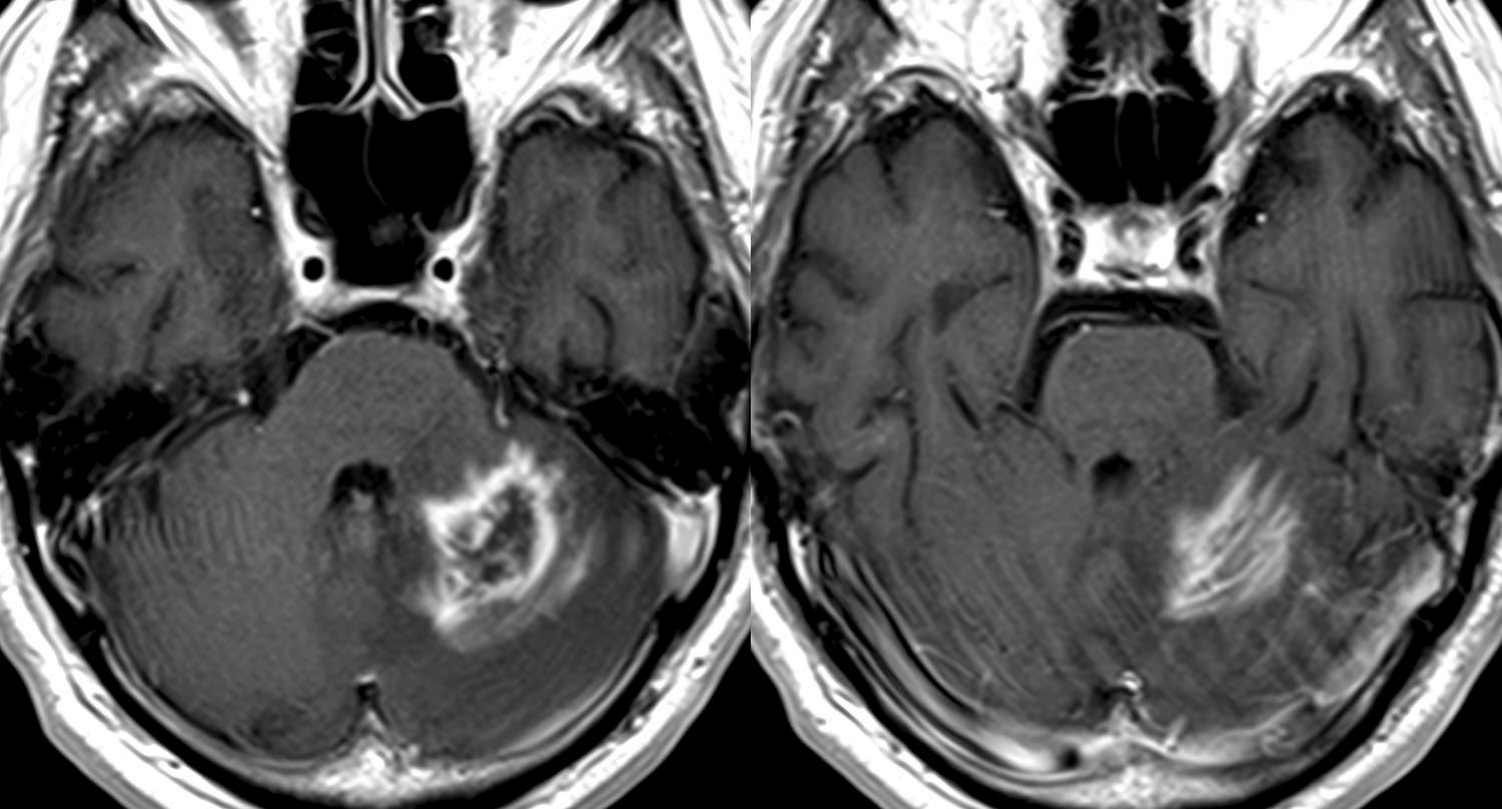
A 70-year-old male patient with radiation necrosis (RN). He had a history of gamma knife surgery for lung cancer metastasis to the cerebellum. The lesion was clinically diagnosed as RN after its size reduced only during the follow-up. The figures show a clear branch-like enhancement on axial two-dimensional contrast-enhanced T1WI. Coronal and sagittal images were not obtained in this case.

**Figure 5:**
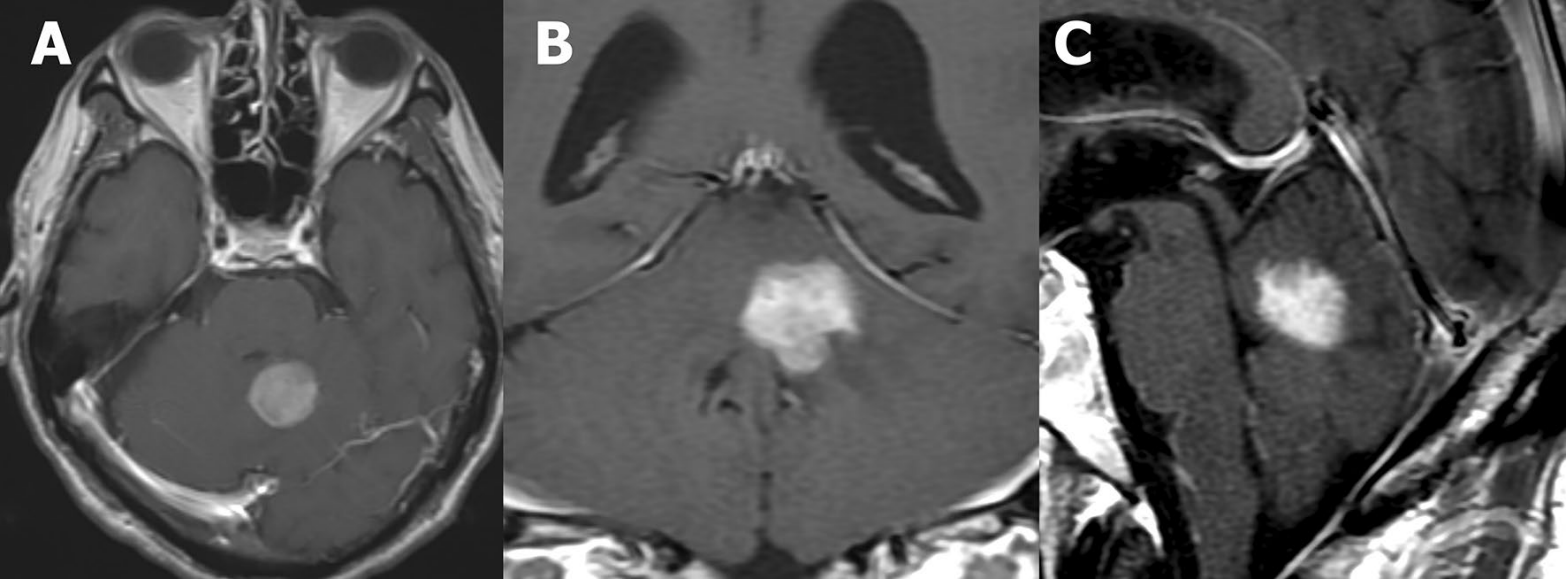
A 54-year-old male patient with primary central nervous system lymphoma. A single enhancing nodule was observed in the left cerebellar hemisphere partly involving the vermis on two-dimensional contrast enhanced T1WI. Branch-like enhancement (BLE) was not observed in the axial (**A**) and coronal (**B**) images. However, moderate BLE was found in the sagittal image (**C**).

BLE on CE-MRI and other imaging findings in each group are shown in Table 2. Of 17 patients with CL, 15 (88%) presented with BLE (Fig. 2). Moreover, only three (5%) of 58 patients in the non-CL group had BLE. One patient newly diagnosed with diffuse midline glioma and one with recurrent anaplastic astrocytoma, both of whom were included in the HGG group, presented with mild BLE in at least one view (Fig. 3). One patient with radiation necrosis who had a history of gamma knife surgery for lung cancer metastasis to the cerebellum had an evident BLE in the axial image (Fig. 4). One-way ANOVA and post hoc testing for pairwise comparisons showed significant differences from the other groups except for radiation necrosis (p < 0.05). The images of all patients, including 15 and 2 who underwent 3D-CE-T1WI and 2D-CE-T1WI, respectively, in the CL group were assessed at multiple views. BLE was not detected in one patient with primary vitreoretinal lymphoma assessed by axial and sagittal images of 2D-CE-T1WI, and in one patient with a non-mass-forming perivascular-enhancing lesion. There was no difference in the presence of BLE and the images with the most visible view of such a finding between evaluators. The weighted kappa values (standard error, 95% confidence interval) of the inter-reader agreement for the degree of BLE were 0.81 (0.077, 0.66–0.96) for axial, 0.89 (0.063, 0.76–1.00) for sagittal, and 0.75 (0.093, 0.57–0.93) for coronal images (Table 3). In patients (n = 16; 14 with CL and 2 with non-CL) who underwent 3D-CE-T1WI, the sagittal images had the most visible BLE (n = 10), followed by the coronal images (n = 6) (Fig. 5).

**Table 2:** Branch-like enhancement and other imaging findings in each group.

	Cerebellar lymphoma	Metastasis	Hemangioblastoma	High grade glioma	Pilocytic astrocytoma	Meduloblastoma	Ependymoma	Radiation necrosis
	(n = 17)	(n = 30)	(n = 12)	(n = 9)	(n = 3)	(n = 2)	(n = 1)	(n = 1)
**Branch-like enhancement**								
Reader1								
Present	15 (88%)	0	0	2 (22%)	0	0	0	1 (100%)
Mild	2 (12%)			2 (22%)				
Moderate	4 (24%)			0				
Clear	9 (53%)			0				
Reader2								
Present	15 (88%)	0	0	2 (22%)	0	0	0	1 (100%)
Mild	2 (12%)			2 (22%)				
Moderate	2 (12%)			0				
Clear	11 (65%)			0				
**Other MRI findings**								
Lesion								
Solitary	9 (53%)	15 (50%)	12 (100%)	2 (22%)	3/3 (100%)	2/2 (100%)	1/1 (100%)	1/1 (100%)
Multiple	8 (47%)	15 (50%)	0	7 (78%)	0	0	0	
Main location								
Hemisphere	9 (53%)	27 (90%)	10 (83%)	8 (89%)	2/3 (67%)			1/1 (100%)
Vermis	6 (35%)	3 (10%)	2 (17%)	1 (11%)	1/3 (33%)	2/2 (100%)	1/1 (100%)	
Tonsil	1 (6%)							
Dendate nucleus	1 (6%)							
DWI high intensity	9/16 (56%)	14/26 (54%)	2/12 (8%)	7/9 (78%)	0/2 (0%)	2/2 (100%)	1//1 (100%)	NA
Low ADC value	13/16 (81%)	12/26 (46%)	0/11 (0%)	5/9 (56%)	0/2 (0%)	2/2 (100%)	1/1 (100%)	NA
Streak-like edema	14/17 (82%)	25/30 (83%)	11/12 (92%)	3/9 (33%)	2/3 (67%)	0/1 (0%)	0/1 (0%)	NA
Cystic component	1/17 (6%)	20/30 (67%)	10/12 (83%)	7/9 (78%)	2/3 (67%)	1/2 (50%)	1/1 (100%)	1/1 (100%)
Hemorrhage	2/17 (12%)	6/28 (21%)	2/12 (17%)	2/9 (22%)	1/3 (67%)	0/2 (0%)	1/1 (100%)	0/1 (0%)
**Other imaging findings**								
CT hyperdensity	13/14 (93%)	21/26 (81%)	7/12 (58%)	3/9 (33%)	2/3 (67%)	1/1 (100%)	1/1 (100%)	0/1 (0%)
Calcification	0/14 (0%)	2/27 (8%)	0/12 (0%)	0/9 (0%)	1/3 (33%)	0/1 (0%)	0/1 (0%)	0/1 (0%)
High FDG uptake	10/11 (91%)	4/12 (33%)	0/1 (0%)	2/5 (40%)	NA	NA	NA	0/1 (0%)

**Table 3:** Inter-reader agreement for BLE in each view.

	Reader 1	Reader 2		*Κ*	95% CI
**BLE in each veiw**					
Axial	12/17 (71%)	12/17 (71%)	Presence of BLE	1	
Mild	5/17 (29%)	4/17 (24%)	Degree of BLE	0.81	(0.66–0.96)
Moderate	4/17 (24%)	3/17 (18%)			
Clear	3/17 (18%)	5/17 (29%)			
Sagital	14/16 (88%)	14/16 (88%)	Presence of BLE	1	
Mild	3/16 (19%)	3/16 (19%)	Degree of BLE	0.89	(0.76–1.00)
Moderate	3/16 (19%)	1/16 (6%)			
Clear	8/16 (50%)	10/16 (63%)			
Coronal	13/16 (81%)	13/16 (81%)	Presence of BLE	1	
Mild	5/16 (31%)	2/16 (13%)	Degree of BLE	0.75	(0.57–0.93)
Moderate	3/16 (19%)	7/16 (44%)			
Clear	5/16 (31%)	4/16 (25%)			

*BLE* branch-like enhancement.

### Other imaging findings

Diffusion restriction was observed in 13 (81%) of 16 patients with CL who had an available ADC map. However, based on a qualitative assessment, only nine (56%) patients had evident hyperintensity on DWI. Diffusion restriction in the non-CL group was not seen in low-grade tumors such as hemangioblastoma and astrocytic glioma. It was seen in nearly half of the cases of metastasis (46%) and HGG (56%), but less frequently than in CL. One-way ANOVA and post hoc testing for pairwise comparison with CL showed only significant differences with hemangioblastoma (p < 0.05). Streak-like edema (Fig. 5) was observed in 14 (82%) of 17 patients with CL and was significantly more common than HGG in three of nine (33%) patients (p < 0.05). However, it was more common in patients with hemangioblastoma (92%) and metastasis (83%), although no significant difference from CL was detected. Patients with CL (6%) rarely presented with a cystic component, but it was usually observed in patients with hemangioblastoma (83%), HGG (78%), and metastasis (63%). However, statistically, pairwise comparisons only showed significant differences with hemangioblastoma. Only two (12%) patients with CL experienced mild hemorrhage. Moreover, hemorrhage was less frequently observed in patients with other conditions without significant difference (9 of 56 evaluable non-CL cases (16%); 4/28 cases of metastasis, 2/12 cases of hemangioblastoma, 2/9 cases of HGG, and 1 case of ependymoma). Meanwhile, none of the patients with pilocytic astrocytoma, medulloblastoma, and radiation necrosis had hemorrhage. A high-density area was observed in 13 (93%) of 14 patients with CL who underwent CT scans. It was seen in 82% of metastases, without significant difference, but in 33% of HGGs, where its frequency was significantly lower than CL (p < 0.05). In benign tumors, it was commonly seen in hemangioblastoma (58%), but less frequent than in CL, although statistically significant differences were not detected. In 10 patients with CL who underwent FDG PET/CT, nine (90%) had a higher uptake in the brain parenchyma. Meanwhile, although the number of cases was small, higher activity than brain parenchyma was observed in two (40%) of five patients with HGG and in 4 (33%) of 12 patients with metastasis. Statistically, a significant difference between metastasis was detected (p < 0.05).

### Comparison between PCNSL and SCNSL

The age, gender, and imaging findings of PCNSL and SCNSL are contrasted in the Table 4. Although the number of cases was too small to show statistically, there was no significant difference between them in this study.

**Table 4:** Comparison between PCNSL and SCNSL.

	PCNSL (n = 12)	SCNSL (n = 5)	*P* value*
Age	65.8 ± 6.4 (54–72)	59.8 ± 25.9 (9–79)	
Female:male	2:10	2:3	0.54
**Branch-like enhancement**			
Reader1			
Present	10 (83%)	5 (100%)	1.00
Mild	2 (17%)	0	
Moderate	3 (25%)	1 (20%)	
Clear	5 (42%)	4 (80%)	
Reader2			
Present	10 (83%)	5 (100%)	1.00
Mild	2 (17%)	0	
Moderate	1 (8%)	1 (20%)	
Clear	7 (58%)	4 (80%)	
**Other MRI findings**			
Lesion			
Solitary	6 (50%)	3 (60%)	1.00
Multiple	6 (50%)	2 (40%)	
Main location			
Hemisphere	5 (42%)	4 (80%)	
Vermis	5 (42%)	1 (20%)	
Tonsil	1 (8%)	0	
Dendate nucleus	1 (8%)	0	
DWI high intensity	5/11 (45%)	4/5 (80%)	0.30
Low ADC value	9/11 (82%)	4/5 (80%)	1.00
Streak-like edema	10/12 (83%)	4/5 (80%)	1.00
Cystic component	1/12 (8%)	0/5 (0%)	1.00
Hemorrhage	1/12 (8%)	1/5 (20%)	0.52
**Other imaging findings**			
CT hyperdensity	9/10 (90%)	4/4 (100%)	1.00
Calcification	0/10 (0%)	0/4 (0%)	1.00
High FDG uptake	8/9 (89%)	2/2 (100%)	1.00

*PCNSL* primary central nervous system lymphoma, *SCNSL* sencoundary CNS system lymphoma, *BLE* branch-like enhancement, * Fisher’s exact test.

## Discussion

This study showed that BLE on CE-MRI is a highly specific finding for CL. Hence, it can be used to effectively distinguish CL from other pathologies. This finding was originally reported by He et al. in 2020 as one of the useful enhancement patterns to differentiate between PCNSL and HGG in the cerebellum^21^. In this study, 8 out of 12 PCNSLs showed BLE, while none of the 15 HGGs showed BLE. Since features such as homogeneous enhancement and less cyst formation contribute more to differentiating PCNSL from HGG in this study, the authors consider BLE to be one of the features to be evaluated comprehensively. It has also been shown that PCNSL has a lower ADC value. However, these have already been reported as features of lymphoma. Therefore, we focused on the novelty of the feature of BLE. A search of the literature for CL with contrast-enhanced MRI images confirms this finding in many cases, even though it has not been reported as BLE^20,23–25^. The earliest report of MRI findings in CL reported an unusual enhancement pattern with detailed comparison to pathology^20^. It is interesting to note that the study by He et al. shows that this finding, which was thought to be unusual 30 years ago, is actually specific.

Although a few patients with HGG presented with mild BLE and one with radiation necrosis had a clear BLE, it was an extremely rare finding in the non-CL group. In patients who underwent 3D-CE-T1WI, BLE was more visible in the sagittal and coronal views than in the axial view. Therefore, BLE in 3D-CE-T1WI images should be assessed cautiously in patients with cerebellar lesions. One patient without BLE on CE-MRI had a non-mass-forming lesion. This lesion was characterized by perivascular enhancement, which is a characteristic of other lymphoproliferative disorders^26^. The findings did not manifest because there was no mass formation, and it was identified at an early stage. The cause of BLE is unknown. The microscopic finding is infiltration of small round cells along the white matter. Nevertheless, the gray matter is preserved^20^, which may be a feature of small round cell tumors. However, in patients with small cell lung cancer, which is also classified as a small round cell tumor and is known to show active behavior^27^, homogeneous enhancement^28^ and low ADC values^29^, their cerebellar metastases did not show BLE. Notably, this finding is rarely observed in glioma, even if the extension along the white matter is a characteristic of this condition^30^. Moreover, the finding was observed in radiation necrosis, which may destroy the existing structure. As there was only one case, further study with a larger number of participants should be performed to assess the frequency of this finding. The peculiarity of the cerebellar structure may be correlated with such a result. Nevertheless, as studies on supratentorial lesions have not yet been performed, this notion has not been validated.

In CL, the common findings include diffusion restriction (81%), high-density areas on a CT scan (93%), and high FDG uptake (90%), and these may be helpful. However, the results were not specific to CL. Streak-like edema, which is useful in differentiating CL from HGG, was observed in 82% of CL cases. Nevertheless, most patients with hemangioblastoma and metastasis presented with this finding.

In this study, all lesions were visually assessed by specialists. Currently, advanced MRI strategies, such as dynamic CE-MRI^31^ and dynamic susceptibility-weighted contrast-enhanced imaging^32^, are the latest and effective techniques used to differentiate CNS lymphoma from other tumors^33^. However, we believe that our results may be helpful in diagnosing CL, which can be identified at an early stage in cases in which multiple pathological conditions are considered. For example, at the time of the initial brain MRI for an unknown pathology, these findings can be observed in patients with a history of cancer and/or systemic lymphoma and immunosuppressive conditions. The non-CL cases in this study included metastasis, primary brain tumors, and radiation necrosis. However, infectious causes, such as tuberculoma, cryptococcoma, pyogenic abscess and cysticercosis, and inflammatory diseases, including tumefactive multiple sclerosis, sarcoidosis, and other granulomatous conditions, were not identified. This is considered a limitation of this single-center analysis; that is, in our institution, cases such as opportunistic infections or rare complications of the disease are rarely encountered. To the best of our knowledge, there are no reports of BLE in these cases. Another limitation is that our research included only DLBCL cases. DLBCL is the most common pathology in CNS lymphoma; however, whether this finding can be observed in other types of lymphoma or lymphoproliferative disorders has not been validated. Furthermore, all patients were negative for the human immunodeficiency virus (HIV), and whether the findings are observed in HIV-related CNS lymphoma is not fully elucidated.

## Conclusion

BLE on CE-MRI is a highly specific finding for CL, both primary and secondary CNS lymphoma. Hence, it is effective in identifying other pathologies in the cerebellum. Moreover, the sagittal 3D-CE-T1WI image is important in evaluating this finding.

## References

[1] Siegel R, Naishadham D, Jemal A (2012). Cancer statistics, 2012. CA Cancer J. Clin..

[2] Montoto S, Lister TA (2005). Secondary central nervous system lymphoma: risk factors and prophylaxis. Hematol. Oncol. Clin. N. Am..

[3] O'Neill BP, Decker PA, Tieu C, Cerhan JR (2013). The changing incidence of primary central nervous system lymphoma is driven primarily by the changing incidence in young and middle-aged men and differs from time trends in systemic diffuse large B-cell non-Hodgkin's lymphoma. Am. J. Hematol..

[4] Villano JL, Koshy M, Shaikh H, Dolecek TA, McCarthy BJ (2011). Age, gender, and racial differences in incidence and survival in primary CNS lymphoma. Br. J. Cancer.

[5] Scott BJ, Douglas VC, Tihan T, Rubenstein JL, Josephson SA (2013). A systematic approach to the diagnosis of suspected central nervous system lymphoma. JAMA Neurol..

[6] Tomita N, Kodama F, Kanamori H, Motomura S, Ishigatsubo Y (2006). Secondary central nervous system lymphoma. Int. J. Hematol..

[7] Fletcher CD, Kahl BS (2014). Central nervous system involvement in diffuse large B-cell lymphoma: an analysis of risks and prevention strategies in the post-rituximab era. Leuk. Lymphoma.

[8] Zhang J, Chen B, Xu X (2014). Impact of rituximab on incidence of and risk factors for central nervous system relapse in patients with diffuse large B-cell lymphoma: A systematic review and meta-analysis. Leuk. Lymphoma.

[9] Suh CH (2014). Atypical imaging features of primary central nervous system lymphoma that mimics glioblastoma: Utility of intravoxel incoherent motion MR imaging. Radiology.

[10] Abrey LE (2005). Report of an international workshop to standardize baseline evaluation and response criteria for primary CNS lymphoma. J. Clin. Oncol..

[11] Valles FE (2013). Combined diffusion and perfusion MR imaging as biomarkers of prognosis in immunocompetent patients with primary central nervous system lymphoma. AJNR Am. J. Neuroradiol..

[12] Zhang Y, Zhang Q, Wang XX, Deng XF, Zhu YZ (2016). Value of pretherapeutic DWI in evaluating prognosis and therapeutic effect in immunocompetent patients with primary central nervous system lymphoma given high-dose methotrexate-based chemotherapy: ADC-based assessment. Clin. Radiol..

[13] Bühring U (2001). MRI features of primary central nervous system lymphomas at presentation. Neurology.

[14] Haldorsen IS, Kråkenes J, Krossnes BK, Mella O, Espeland A (2009). CT and MR imaging features of primary central nervous system lymphoma in Norway, 1989–2003. AJNR Am. J. Neuroradiol..

[15] Cheng G, Zhang J (2019). Imaging features (CT, MRI, MRS, and PET/CT) of primary central nervous system lymphoma in immunocompetent patients. Neurol. Sci.

[16] Haldorsen IS, Espeland A, Larsson E-M (2011). Central nervous system lymphoma: Characteristic findings on traditional and advanced imaging. Am. J. Neuroradiol..

[17] Wu Y (2019). Parenchymal central nervous system involvement in aggressive B-cell lymphoma: Retrospective analysis of clinical and MRI features in a Chinese population. BMC Neurol..

[18] Koubska E, Weichet J, Malikova H (2016). Central nervous system lymphoma: A morphological MRI study. Neuro Endocrinol. Lett..

[19] Malikova H (2018). Secondary central nervous system lymphoma: Spectrum of morphological MRI appearances. Neuropsychiatr. Dis. Treat..

[20] Gupta RK (1993). Unusual enhancement on gadolinium-enhanced MRI in a case of primary cerebellar lymphoma. Neuroradiology.

[21] He YX, Qu CX, He YY, Shao J, Gao Q (2020). Conventional MR and DW imaging findings of cerebellar primary CNS lymphoma: Comparison with high-grade glioma. Sci. Rep..

[22] Oyama J (2021). Incidental T2 hyperintensities in the medial part of the bilateral globus pallidus are possibly an age-related physiological finding. Neuroradiol. J..

[23] Beraldo GL (2019). Primary infratentorial diffuse large b-cell lymphoma: A challenging diagnosis in an immunocompetent patient. Rev. Assoc. Med. Bras..

[24] Yamamoto J, Kitagawa T, Akiba D, Nishizawa S (2015). 5-Aminolevulinic acid-induced fluorescence in cerebellar primary central nervous system lymphoma: A case report and literature review. Turk. Neurosurg..

[25] Fløisand Y (2011). Richter syndrome presenting as a solitary cerebellar tumor during first-line treatment for chronic lymphocytic leukemia. Leuk. Lymphoma.

[26] Pittock SJ (2010). Chronic lymphocytic inflammation with pontine perivascular enhancement responsive to steroids (CLIPPERS). Brain J. Neurol..

[27] van Meerbeeck JP, Fennell DA, De Ruysscher DK (2011). Small-cell lung cancer. Lancet.

[28] Xu X (2019). Clinical utility of quantitative dual-energy CT iodine maps and CT morphological features in distinguishing small-cell from non-small-cell lung cancer. Clin. Radiol..

[29] Jung WS, Park CH, Hong CK, Suh SH, Ahn SJ (2018). Diffusion-weighted imaging of brain metastasis from lung cancer: Correlation of MRI parameters with the histologic type and gene mutation status. AJNR Am. J. Neuroradiol..

[30] Morales La Madrid A, Ranjan S, Warren KE (2018). Gliomatosis cerebri: a consensus summary report from the Second International Gliomatosis cerebri Group Meeting, June 22-23, 2017, Bethesda, USA. J. Neuro-oncol..

[31] Fu F (2021). Dynamic contrast-enhanced magnetic resonance imaging biomarkers predict chemotherapeutic responses and survival in primary central-nervous-system lymphoma. Eur. Radiol..

[32] Kickingereder P (2014). Primary central nervous system lymphoma and atypical glioblastoma: multiparametric differentiation by using diffusion-, perfusion-, and susceptibility-weighted MR imaging. Radiology.

[33] Suh CH (2019). MRI as a diagnostic biomarker for differentiating primary central nervous system lymphoma from glioblastoma: A systematic review and meta-analysis. J. Magn. Resonance Imaging.

